# Implications of peripapillary retinal nerve fiber layer thickening by optical coherence tomography in children with pars planitis

**DOI:** 10.1186/s12348-025-00564-9

**Published:** 2025-12-10

**Authors:** Diala Abu Al-Halawa, Radgonde Amer

**Affiliations:** 1 Faculty of Medicine, Hebrew University of Jerusalem, Jerusalem, Israel; 2 Department of Ophthalmology, Hadassah Medical Center, Jerusalem, 91120 Israel

**Keywords:** Pars planitis, Retinal nerve fiber layer thickness, Optical coherence tomography, Intermediate uveitis in children, Binocular indirect ophthalmoscopy score, Papillitis

## Abstract

**Purpose:**

To assess retinal nerve fiber layer thickness by optical coherence tomography (OCT-RNFLT) in children with pars planitis and to examine its associations with visual acuity, binocular indirect ophthalmoscopy score (BIO), ocular signs and medical therapies.

**Methods:**

This was a retrospective study. Demographic, clinical and imaging data were collected.

**Results:**

Included were 29 children (50 eyes) with a mean age of 7.9 ± 2.9 years. Presenting OCT-RNFLT (28 eyes) was 209.6 ± 179 μm. Twenty-two eyes (78.6%) had OCT-RNFLT ≥ 130 μm and it was significantly associated with higher BIO score (2.02 ± 1.21 vs. 0.42 ± 0.80 in eyes with OCT-RNFLT < 130 μm), anterior uveitis, papillitis, conventional disease-modifying antirheumatic drugs and biologic therapy. Following the initiation of therapy, improvement in BIO score was observed promptly, preceding the improvement in OCT-RNFLT. In the univariate general linear model, BIO group classification demonstrated the strongest association with RNFLT (*F* = 16.69, *p* < 0.001). Papillitis was also significantly associated with higher RNFL values (*F* = 6.47, *p* = 0.015). Anterior uveitis did not show a significant association with RNFLT (*F* = 0.02, *p* = 0.883), suggesting that posterior segment findings could be more relevant indicators of RNFLT in this cohort.

**Conclusion:**

To date, this is the first longitudinal study that investigated OCT-RNFLT in pediatric pars planitis patients. OCT-RNFLT strongly correlated with disease severity and eyes with OCT-RNFLT *≥*130 µm required at a higher rate the institution of intensive immunosuppressive regimen. The reduction in OCT-RNFLT with resolving inflammation highlights its potential role as a tool for monitoring therapeutic response over time.

## Backgound

Pars planitis is a chronic inflammatory process of the ciliary body and the vitreous is the predominant site of inflammation [1, 2]. It is an idiopathic condition that develops in the absence of an underlying infectious or systemic inflammatory disease [2]. It is characterized by the formation of vitreous snowballs and snowbanking. It commonly affects children and adolescents and is the second most common form of uveitis in childhood [3, 4].

Children are commonly asymptomatic [5] and may thus present late due to parents’ unawareness and the lack of external signs of inflammation such as redness. Diagnosing pediatric uveitis may therefore be challenging. Insufficient time may be available to ophthalmologists to perform a complete eye examination because of limited patients’ cooperation. In addition, the inability to perform detailed imaging studies such as fluorescein angiography (FA) in children who are anxious or photophobic may further delay prompt assessment of uveitis and its complications.

Optical coherence tomography (OCT) is a feasible, rapid, non-invasive imaging modality that is commonly in use in ophthalmology clinics. It provides a thorough “in-vivo” assessment of the retinal layers as it allows the detection of fine morphological changes in retinal microstructure [6]. This has improved the diagnostic evaluation and decision-making abilities of ophthalmologists [6].

Recently, measuring retinal nerve fiber layer thickness on optical coherence tomography (OCT-RNFLT) was shown to be a useful tool in the diagnosis of papillitis in children with uveitis with relatively high sensitivity and specificity [7].

Another publication reported that uveitic eyes with active inflammation had a significant thickening of the RNFL measurements in comparison to eyes with inactive uveitis [8].

We therefore aimed to assess OCT-RNFLT in pediatric pars planitis patients and to study its associations with visual acuity, degree of vitreous haze, ocular signs and complications and medical therapies during the follow-up period.

## Methods

This was a retrospective study of consecutive patients with pars planitis who were diagnosed at ≤ 18 years of age. Data were collected from the medical records of patients between 2010 and 2023. Diagnosis of pars planitis was in accordance with the criteria of the Standardization of Uveitis Nomenclature (SUN) workgroup [2]. All children were evaluated by a pediatric rheumatologist and systemic inflammatory conditions were ruled-out. Patients with a follow-up period of less than 6 months were excluded. The study was approved by the ethics review committee (No. 0575–12-HMO) and included waiver of informed consent for the chart review. The study adhered to the tenets of the declaration of Helsinki.

Clinical information that was collected included age at diagnosis, gender, ocular and medical history, ocular complaints recorded at the initial visit and their duration, follow-up time and medical treatment.

Best-corrected visual acuity (BCVA) was assessed using the Snellen chart at presentation and at 1, 3, 6, 12, 24 and 36 months. LogMAR (logarithm of the minimum angle of resolution) notation was used to compute the change in visual acuity (VA).

The clinical signs that were recorded included laterality, anterior uveitis, papillitis, snowbanking, peripheral perivascular sheathing and binocular indirect ophthalmoscopy (BIO) score (vitreous haze) which was ranked using an ordinal scale ranging from 0 to 4 + according to Nussenblatt et al [9]. Anterior segment complications like corneal endotheliopathy, band keratopathy, posterior synechiae and cataract were recorded. Papillitis was defined as a hyperemic optic disc (OD) ± edema in the absence of afferent pupillary defect. Cystoid macular edema (CME) was defined as the presence of intraretinal and/or subretinal fluid on optical coherence tomography (OCT). Ocular hypertension (intraocular pressure > 21 mmHg) and signs of glaucomatous optic neuropathy were also recorded.

Diagnostic investigations included baseline tests of complete blood count, kidney and liver function tests, erythrocyte sedimentation rate (ESR), C-reactive protein (CRP) levels and chest X-ray. Additional tests were tailored based on patients’ medical history.

Ocular imaging was performed, including optical coherence tomography (OCT) (Heidelberg Spectralis OCT, Heidelberg Engineering, Heidelberg, Germany), color fundus photography (Optos Silverstone swept-source OCT, Optos PLC, Dunfermline, UK and TRC-50DX, Topcon Corporation/Kabushiki-gaisha Topukon, Tokyo, Japan) and fundus fluorescein angiography (FFA) (Optos Silverstone swept-source OCT, Optos PLC, Dunfermline, UK).

OCT-RNFL thickness was measured on a cross-sectional retinal image consisting a 3.4-mm diameter circle centered on the OD. Each B-scan consisted of 256 individual A-scans along a circular scan path. The average of 3 B-scans was used in the analysis. Mean RNFL thickness in micrometers (µm) along the whole circle circumference, four quadrants, 12 o’clock hours and at 256 A-scan lengths was obtained [10]. OCT measurements of RNFL thickness for patients were done on the same machine. Images with poor centration or poor quality (< 15 dB) were excluded from analysis. All segmentations of OCT-RNFL were checked by the authors and inaccuracies in the automated segmentation were corrected manually.

Systemic treatment was initiated in patients who have one or more of the following: vitritis resulting in reduced vision, vitreous haze (BIO score) > 1, CME documented by OCT, macular leakage by fluorescein angiography.

Medical therapy recorded included systemic corticosteroids, conventional disease-modifying antirheumatic drugs (cDMARDs) and biologic agents at the prespecified time points. Adalimumab (Humira®; AbbVie Inc., Ludwigshafen, Germany) was instituted at the dose of 40 mg subcutaneously and repeated every other week in patients weighing ≥ 30 kg. In children weighing < 30 kg, adalimumab was instituted at the dose of 20 mg subcutaneously and repeated every other week. Serological tests for hepatitis B and hepatitis C, Mantoux test ± QuantiFERON test were obtained before institution of biologic treatment. Screening brain magnetic resonance imaging (MRI) was performed before commencing a subject on anti-tumor necrosis factor (TNF)-α drugs, as these agents have a risk of worsening demyelinating disease [11].

Since the ideal cut-off of OCT-RNFLT for diagnosing papillitis was *>*130 µm in a recent publication in children with uveitis [7], we aimed to study the associations between OCT-RNFLT ≥ 130 µm and presenting ocular signs and complications, the presence of angiographic OD and macular leakage and the different types of systemic medical therapies. We further aimed to assess the trends of BCVA, BIO score and OCT-RNFLT changes over the first 12 months following presentation and to examine if associations existed between those three parameters.

## Statistical analysis

Statistical analyses were performed with the SPSS statistical package, version 24 (SPSS Inc., Chicago, IL). Continuous variables are presented as mean and standard deviation (SD); categorical variables are presented as frequencies and percentages. In order to test the associations between two categorical variables, the chi-square or Fisher’s exact test was used.

Student’s T test was used to analyze the difference between logMAR BCVA at presentation and at the follow-up visits in all the eyes, and to compare OCT-RNFL thickness and BIO score at the specific time points. All statistical tests applied were two-tailed and a p value of 5% or less was considered statistically significant. ANOVA was used to test for significant associations between OCT-RNFL and other study variables.

In order to incorporate BIO score in univariate analysis model, BIO score was categorized into three categories based on the frequency of the distribution to:(A) zero, (B) 0.5 to 2.5, (C) 3 and above. Univariate general linear model was used to evaluate disease severity prediction of OCT-RNFL thickness. A p value of the model of 5% or less was considered statistically significant. To address the imbalance in the distribution of RNFL thickness and to enhance the generalizability of our findings, we applied the Synthetic Minority Over-sampling Technique (SMOTE) [12]. This technique balances the dataset by generating synthetic examples for the minority class while removing ambiguous or noisy samples from the majority class, thereby creating a more robust dataset for predictive modeling. After balancing the RNFL thickness variable by 130 µm using SMOTE analysis, the final number of cases was 44: 22 cases in each group.

## Results

### Demographic characteristics and presenting symptoms

Included were 29 children, with a mean ± SD age at diagnosis of 7.9 ± 2.9 years. Boys were predominantly affected (20/29, 69%). It was a bilateral disease in 21 patients (72.4%), thus yielding 50 affected eyes. The mean ± follow-up time was 47.5 ± 39.1 months. Ten patients (34.5%) were asymptomatic and were diagnosed to have pars planitis on routine eye examination (Table 1). In the remaining 19 patients, the presenting complaints were drop in vision in 19 eyes, blurred vision in 3 eyes, floaters in 8 eyes and pain in 1 eye. In symptomatic patients, the mean ± SD duration of complaints was 12.8 ± 17.5 weeks: it was 1–4 weeks in 8 patients and > 12 weeks in 8 patients. In three patients, symptoms’ duration was 5–12 weeks.

**Table 1:** Demographic features, laterality, follow-up time, duration of complaints, number of asymptomatic patients and medical therapies and their duration

Number of patients (eyes)	29 (50 eyes)
Boys: Girls	20 (69%):9 (31%)
Mean age ± SD at presentation	7.9 ± 2.9 years
Laterality of pars planitis	Bilateral in 21 patients: unilateral 8 patients
Mean Follow-up time (months)	47.5 ± 39.1
Mean ± SD duration of complaints (weeks)	12.8 ± 17.5 weeks
Pars planitis discovered on routine exam	10 patients (34.5%)
**Medical therapies and their mean ± SD duration in months**	
Systemic steroids	24 patients (82.8%)- 22.7 ± 26 months
Conventional disease-modifying antirheumatic drugs	28 patients (96.6%)-34.4 ± 27.9 months
Biologic therapy	15 patients (51.7%)-14.2 ± 18.9 months
Observation only	1 patient (3.4%)

## Ocular features

**Fig. 1:**
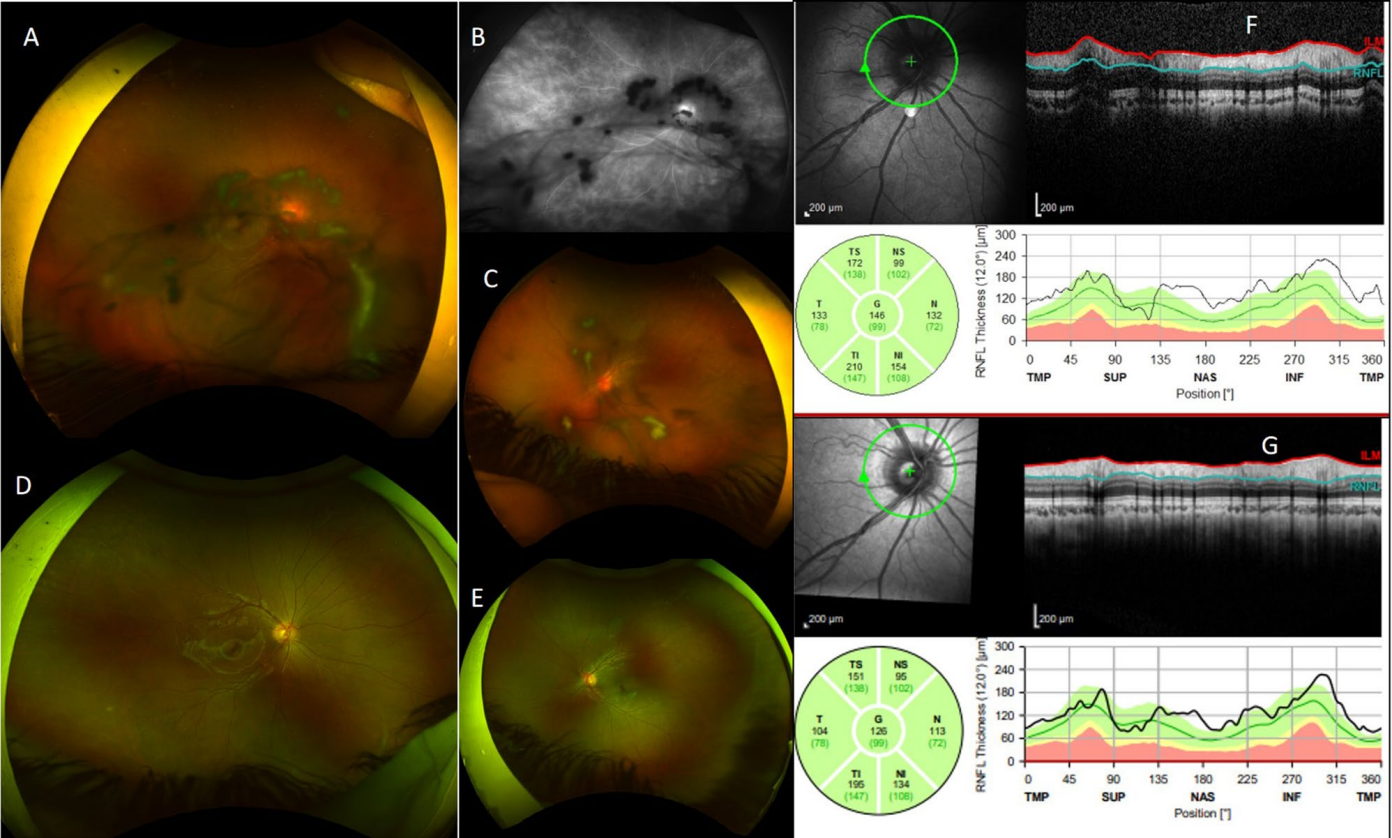
**A**-Ultra-wide field fundus photograph of right eye at presentation shows marked vitreous opacities and snowballs covering the posterior pole and inferior fundus. **B**-Ultra-wide field fundus fluorescein angiography of right eye at presentation (late-phase) shows marked diffuse capillary hyperfluorescence and optic disc hyperfluorescence. **C**-Ultra-wide field fundus photograph of left eye at presentation shows snowballs in the vitreous cavity. **D**, **E**-Ultra-wide field fundus photographs of right and left eyes respectively three years following presentation show marked improvement of vitreous opacities and snowballs. **F**-Spectral-domain optical coherence tomography of the right eye at presentation shows marked retinal nerve fiber layer thickening at 146 µm and in G-marked improvement is noted three years later with a thickness of 125 µm

The three most common ocular signs that were diagnosed at presentation included anterior uveitis in 20 eyes (40%), papillitis in 14 eyes (28%) and snowbanking in 11 eyes (22%) (Table 2, Fig. 1). Five eyes (10%) had peripheral vascular sheathing. Corneal endotheliopathy, band keratopathy, posterior synechaie and cataract were detected in 10 (20%), 6 (12%), 4 (8%) and 3 (6%) eyes respectively. With regard to posterior segment complications, nine eyes (18%) were diagnosed to have CME by SD-OCT (mean ± SD central macular thickness of 484.7 ± 126.8 µm). Vitreous hemorrhage at presentation was diagnosed in one eye (2%).

**Table 2:** LogMAR best-corrected visual acuity, binocular indirect ophthalmoscopy score and retinal nerve fiber layer thickness by optical coherence tomography at presentation, ocular signs and complications at presentation and ocular complications during follow-up time and fluorescein angiographic features at presentation

Mean ± SD LogMAR BCVA at presentation	0.47 ± 0.6
Mean ± SD BIO score at presentation	1.7 ± 1.4
Mean RNFL thickness by OCT at presentation	209.6 ± 88.1 μm
**Ocular signs and complications at presentation (number of eyes, percentage)**	
Corneal endotheliopathy	10 (20%)
Band keratopathy	6 (12%)
Posterior synechiae	4 (8%)
Cataract	3 (6%)
Anterior uveitis	20 (40%)
Papillitis	14 (28%)
Peripheral perivascular sheathing	5 (10%)
Snowbanking	11 (22%)
Vitreous hemorrhage	1 (2%)
Cystoid macular edema on OCT	9 (18%)
**Ocular complications during follow-up (number of eyes, percentage)**	
Vitreous hemorrhage	1 (2%)
Retinal Detachment	1 (2%)
Cystoid macular edema on OCT	3 (6%)
**Fluorescein angiographic features at presentation** **(number of eyes/interpretable FA images and percentage)**	
Peripheral vascular leakage	26/26 eyes (100%)
Optic disc leakage	20/25 (80%)
Macular leakage	15/24 (62.5%)

BCVA:best-corrected visual acuity, BIO: binocular indirect ophthalmoscopy, RNFL: retinal nerve fiber layer, OCT: optical coherence tomography

FFA was performed in 16 patients (26 affected eyes) at presentation. All eyes showed peripheral vascular leakage. Optic disc and macular leakage were noted in 20/25 eyes (80%) and 15/24 eyes (62.5%) respectively (it was not possible to decide on OD and macular leakage in one eye and 2 eyes respectively because of dense vitreous opacities obscuring the view of the posterior pole) (Fig. 1).

**Fig. 2:**
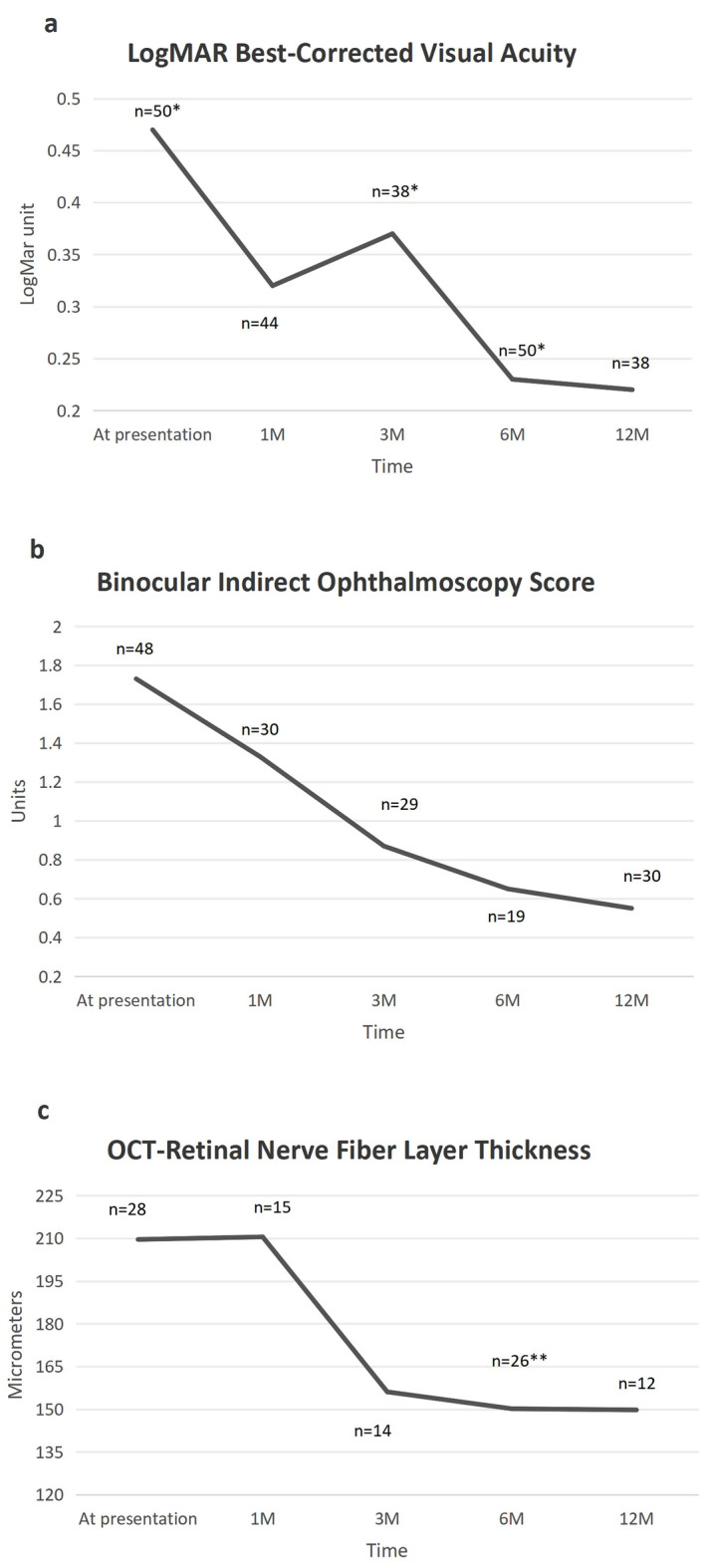
Figure 2**a** demonstrates LogMAR best-corrected visual acuity at presentation and at one, three, six and 12 months of follow-up. Figure 2b demonstrates binocular indirect ophthalmoscopy score at presentation and at one, three, six and 12 months of follow-up. Figure 2c demonstrates retinal nerve fiber layer thickness on optical coherence tomography at presentation and at one, three, six and 12 months of follow-up. Missing data possibly led to inaccuracies but the study results demonstrated a clear trend of improvement in LogMAR BCVA, BIO score and OCT-RNFLT over time

The mean ± SD presenting LogMAR BCVA was 0.47 ± 0.6. It was 0.32 ± 0.34,0.37 ± 0.35,0.23 ± 0.24 and 0.22 ± 0.33 at one, three, six and 12 months respectively (Fig. 2a). The only statistically significant improvement in BCVA was between presentation and 12 months (p = 0.024).

*indicates the time points at which logMAR BCVA was significantly associated with BIO score (at presentation and at 3 and 6 months of follow-up), **indicates the time point at which OCT-RNFL thickness was significantly associated with BIO score (at 6 months of follow-up).

The mean ± SD presenting BIO score was 1.73 ± 1.4. It progressively improved over the follow-up period to 1.33 ± 1.08, 0.87 ± 0.82, 0.65 ± 0.82 and 0.55 ± 0.72 at one, three, six and 12 months respectively (p < 0.05 between presentation and all the follow-up time points) (Fig. 2b).The decrease in BIO score was also significant between months one and three (p = 0.005).

The mean ± SD OCT-RNFLT at presentation was 209.6 ± 88 µm and no change was noted one month later (210.5 ± 112.6 µm). It improved to 156.1 ± 22.9 µm, 150.2 ± 34.5 µm and 149.8 ± 23.7 µm at three, six and 12 months, respectively (p < 0.05 between presentation and 3, 6 and 12 months) (Fig. 2c).

BIO scores improved promptly after therapy initiation, preceding the improvement in OCT-RNFL measurements. BIO score showed a significant decrease one month after starting therapy, whereas OCT-RNFL first exhibited significant improvement three months into therapy. Notable improvement in BCVA subsequently followed, at 12 months into therapy (Fig. 2).

During follow-up, CME was diagnosed in 3 eyes (6%), exudative retinal detachment and vitreous hemorrhage were diagnosed in one eye each (2%).

## Medical therapy

28 patients (96.6%) were treated with methotrexate, 24 patients (82.8%) were treated with prednisone and 15 patients (51.7%) were treated with TNF-ɑ blockers (Adalimumab in 14 patients and infliximab in one patient). Mean duration of therapy was 34.4 ± 27.9, 22.7 ± 26 and 14.2 ± 18.9 months respectively (Table 1). Three patients (10.3%) were treated with monotherapy, 11 (37.9%) with duotherapy and 14 (48.3%) with triple therapy. One patient was observed only and did not receive medical therapy because of the mild degree of the inflammation and the absence of ocular complications.

### Association between OCT-RNFL thickness and demographic, clinical, angiographic features and medical therapies

In order to study the diagnostic implications of thickened OCT-RNFL, we stratified the eyes into two groups 

(Table 3): Eyes with OCT-RNFLT ≥ 130 µm and eyes with OCT-RNFLT < 130 µm.

**Table 3:** Association between clinical variables, medical therapies and retinal nerve fiber thickness by optical coherence tomography (<130 μm vs ≥ 130 μm)

Variable	RNFL Thickness < 130 μm at presentation (Number of eyes (%))	RNFL Thickness ≥ 130 μm at presentation (Number of eyes (%))	F-value	Significance
**LogMAR BCVA (Mean ± SD)**	0.28 ± 0.33/ 6 (21.4)	0.51 ± 0.63/ 22 (78.6)	0.733	0.4
**BIO score (Mean ± SD)**	0.42 ± 0.80/ 6 (21.4)	2.02 ± 1.21/ 22 (78.6)	9.313	0.005
**Endotheliopathy (28 eyes)**			1.639	0.212
No	6 (26.1)	17 (73.9)		
Yes	0 (0.0)	5 (100.0)		
**Band Keratopathy (28 eyes)**			0.557	0.462
No	6 (23.1)	20 (76.9)		
Yes	0 (0.0)	2 (100.0)		
**Posterior Synechiae (28 eyes)**			0.88	0.357
No	6 (24.0)	19 (76.0)		
Yes	0 (0.0)	3 (100.0)		
Cataract (26 eyes)*				p not applicable
No	6 (23.1)	20 (76.9)		
Yes	0 (0.0)	0 (0.0)		
**Anterior uveitis (28 eyes)**			4.643	0.041
No	6 (33.3)	12 (66.7)		
Yes	0 (0.0)	10 (100.0)		
Papillitis (26 eyes)*			4.531	0.044
No	6 (35.3)	11 (64.7)		
Yes	0 (0.0)	9 (100.0)		
**CME on OCT (28 eyes)**			2.6	0.119
No	6 (28.6)	15 (71.4)		
Yes	0 (0.0)	7 (100.0)		
Peripheral Perivascular Sheathing (26 eyes)*			1.385	0.251
No	6 (27.3)	16 (72.7)		
Yes	0 (0.0)	4 (100.0)		
Snowbanking (26 eyes)*			0.615	0.44
No	6 (25.0)	18 (75.0)		
Yes	0 (0.0)	2 (100.0)		
**Optic leakage on FA (18 eyes)**			0.018	0.896
No	1 (20)	4 (80)		
Yes	3 (23.1)	10 (76.9)		
**Systemic Steroids (28 eyes)**			0.265	0.611
No	2 (28.6)	5 (71.4)		
Yes	4 (19.0)	17 (81.0)		
**cDMARDS (28 eyes)**			10.214	0.004
No	2 (100.0)	0 (0.0)		
Yes	4 (15.4)	22 (84.6)		
**Biologic Therapy (28 eyes)**			6.686	0.016
No	6 (37.5)	10 (62.5)		
Yes	0 (0.0)	12 (100.0)		

BCVA: best-corrected visual acuity, BIO: binocular indirect ophthalmoscopy, RNFL: retinal nerve fiber layer, CME: cystoid macular edema, OCT: optical coherence tomography, FA:fluorescein angiography, cDMARDS: conventional disease-modifying antirheumatic drugs. *missing information from medical files or not possible to decide on the presence of the ocular sign because of dense vitreous opacities obscuring the view of the retina

OCT-RNFLT measurements at presentation were available for 28 eyes. Of these, 22 eyes (78.6%) had an OCT-RNFLT of ≥ 130 µm. These eyes demonstrated a higher mean BIO score (2.02 ± 1.21) compared with eyes that had an OCT-RNFLT < 130 µm (0.42 ± 0.80). This difference was statistically significant (p = 0.005). OCT-RNFLT ≥ 130 µm at presentation was significantly associated with anterior uveitis and papillitis (p = 0.041 and p = 0.044 respectively). Furthermore, a significant association was demonstrated between OCT-RNFLT ≥ 130 µm and cDMARDs and biologic therapy (p = 0.004 and p = 0.016 respectively) (Table 3). No significant association was elicited between OCT-RNFLT *≥*130 µm and LogMAR BCVA or CME.

### Univariate general linear model

A univariate general linear model was used to examine the association between clinical predictors that were found to be significantly associated with increased RNFLT (defined as ≥ 130 µm) by ANOVA analysis on Table 3, using the balanced RNFL variable. The overall model was statistically significant (*F*(3, 38) = 15.11, *p* < 0.001), explaining 51% of the variance in RNFL status (adjusted R² = 0.508). Among the variables assessed, BIO group classification demonstrated the strongest association with RNFL thickening (*F* = 16.69, *p* < 0.001) (Table 4). The presence of papillitis in the affected eye was also significantly associated with higher RNFL values (*F* = 6.47, *p* = 0.015). In contrast, anterior uveitis did not show a significant association with RNFL status (*F* = 0.02, *p* = 0.883), suggesting that posterior segment findings could be more relevant indicators of RNFLT in this cohort.

**Table 4:** Univariate general linear model: predictors of high RNFL thickness using balanced variable

Source	Type III Sum of Squares	df	Mean Square	F-value	Significance
**Corrected Model**	5.699^a^	3	1.900	15.110	.000
**Intercept**	0.355	1	0.355	2.824	.101
**BIO_GROUPS**	2.098	1	2.098	16.685	.000
**Anterior uveitis**	0.003	1	0.003	0.022	.883
**Papillitis**	0.813	1	0.813	6.468	.015
**Error**	4.777	38	0.126		
**Total**	20.000	42			
**Corrected Total**	10.476	41			

^a^ R² = 0.544 (Adjusted R² = 0.508)

## Discussion

In the present study, we evaluated the diagnostic and therapeutic implications of OCT-RNFL thickening in pediatric pars planitis patients. OCT-RNFLT was markedly increased on first presentation. We identified a significant and consistent association between the magnitude of OCT-RNFLT and the grade of BIO score (which is a qualitative indicator of disease severity). OCT-RNFLT ≥ 130 µm was observed in eyes with more severe vitritis prompting the need for an intensive regimen of anti-inflammatory therapies.

Similarly, Kouwenberg et al [7] reported that OCT-RNFLT *>*130 µm in children with uveitis was linked to a higher prevalence of active inflammation (OR 4.3). Moore DB et al [8] found that eyes with active uveitis exhibited higher OCT-RNFL measurements compared to eyes with inactive uveitis (140.5 vs. 107.8 µm). Lee MW et al [13] observed in eyes with acute anterior uveitis that OCT-RNFLT tended to increase with greater anterior chamber inflammation.

Moreover, we observed a positive association between OCT-RNFLT *≥*130 µm at presentation and papillitis. This finding aligns with a previous study [7] where OCT-RNFLT *>*130 µm was associated with a higher prevalence of OD swelling (OR 13.7). Papillitis in children with intermediate uveitis holds a significant prognostic value as it is strongly linked to the development of CME (OR 12.4) [5]. Similarly, in children with juvenile idiopathic arthritis-associated uveitis, Tappeiner C et al [14] reported that OD swelling at baseline indicated an increased risk for subsequent CME manifestation (hazard ratio (HR) 2.81). The authors reported that OD swelling was present in 13.8% of eyes six months prior to CME diagnosis and it developed in 48.8% of eyes at the time of CME onset [14].

In our analysis, no significant association was elicited between OCT-RNFLT *≥*130 µm and the development of CME. Conversely, Kouwenberg et al [7]. reported a significant association between OCT-RNFL *>*130 µm and CME (OR 5.3). In the former study [7], 57.5% of the eyes that underwent both OCT-RNFL and FA had panuveitis while only 26.3% had intermediate uveitis. This finding reflects a greater severity of uveitis in their cohort which could explain the higher occurrence of CME observed. In our study, all eyes with CME had OCT-RNFLT *≥*130 µm; however, this association did not reach statistical significance. We speculate that the lack of significance in our analysis may be due to the low proportion of eyes with CME at presentation.

Papillitis in intermediate uveitis is known to be associated with worse VA at presentation [5, 15, 16]. In the current study, eyes with OCT-RNFLT *≥*130 µm demonstrated a trend toward worse presenting BCVA. Although this trend did not reach statistical significance, it remains clinically meaningful. Studies with a large sample size may yield different results; thus, highlighting the need for further investigation in this area.

In our analysis, a positive association was observed between OCT-RNFLT *≥*130 µm and the presence of anterior uveitis. This association may reflect a more severe form of pars planitis with a higher likelihood of subsequent complications. Holland GN et al [17], reported that papillitis, increased anterior chamber cells and flare at baseline predicted new complications in children with chronic anterior uveitis. In the same cohort, papillitis and increased flare were also among the factors that predicted vision loss.

We observed that patients with OCT-RNFLT *≥*130 µm required at a higher rate the institution of second-line steroid-sparing agents and TNF-ɑ blockers. This finding suggests that OCT-RNFLT *≥*130 µm can be considered as a severity index, predicting the need for a more intensive regimen of immunosuppressive medications. Prompt and combination medical therapies led to marked improvement in BIO score and effectively limited the development of ocular complications. Only 10% of eyes sustained new ocular complications over the follow-up period.

Kouwenberg et al [7] reported that OCT-RNFLT *>*130 µm was particularly prevalent in eyes with intermediate and panuveitis, observed in 72% and 45% of the cases, respectively. In the same study, eyes with intermediate uveitis had the highest RNFL thickness (133 µm), followed by those with panuveitis (118 µm) and anterior uveitis (108 µm). Their findings are comparable to ours, as 78.6% of eyes at presentation exhibited OCT-RNFLT *≥*130 µm. In contrast, Moore et al [8] did not identify a correlation between the anatomical type of uveitis (anterior, intermediate, or posterior) and the global RNFLT. Similarly, Gutiérrez-Ezquerro R et al [18] did not find a link between the degree of RNFLT and the anatomical type of uveitis. However, their study did show a trend toward higher mean RNFL values in posterior uveitis compared to anterior uveitis (142.38 vs 123.88 µm). This observation may be influenced by the small proprotion of patients with posterior segment inflammation as the majority of cases (55.2%) had anterior uveitis.

In the study by Kouwenberg et al [7], eyes with papillitis (based on FA) had significantly higher OCT-RNFLT compared to eyes without papillitis (148 vs 109 µm). This distinction was not observed in our cohort. We speculate that the lack of correlation is possibly due to the high prevalence of OD leakage on FA across the majority of eyes in the present study, which may have limited the ability to detect a statistically significant difference.

The fact that BIO scores improved rapidly after one month, while OCT-RNFLT only showed significant improvement after three months, suggests that BIO score may be a more immediate indicator of ocular inflammation, while OCT-RNFL changes reflect more chronic structural changes. The BIO score progressively decreased over time, thus representing a clinical measure of disease control, with the greatest improvements seen in the first three months of therapy. Prior publications reported that RNFL thickness did not return to normal values in some patients with uveitis, even when the rest of the inflammation parameters did [8, 18].

Ophthalmologists should be aware that OCT-RNFL thickening in eyes with active uveitis may mask underlying RNFL thinning, a critical parameter for monitoring glaucoma progression. Din et al [19]. recommended screening for glaucomatous RNFL thinning using OCT during periods of uveitis quiescence to minimize the masking effect of RNFL thickening associated with active uveitis. In patients with Behçet disease (BD) [20], eyes with ocular involvement exhibited significant RNFL thinning compared to eyes without ocular involvement. This thinning is attributed to occlusive vasculitis and subsequent impairment of perfusion. Similarly, patients with systemic lupus erythematosus (SLE) showed notable thinning in the RNFL compared to healthy controls with studies reporting progressive RNFL thinning after a one-year follow-up period [21]. Therefore, OCT-RNFL measurements in eyes affected by glaucoma, Behçet uveitis or SLE may be misleading, potentially resulting in incorrect clinical interpretations and treatment decisions.

Prostaglandins and inflammatory cytokines play a key role in the pathogenesis of uveitic macular edema by increasing the permeability of blood– aqueous and blood–retina barriers. This promotes leukocyte migration and the accumulation of fluid within the retina and subretinally. It is speculated that these same inflammatory mediators contribute to RNFL thickening observed in eyes with uveitis [22, 23].

A limitation of this study is its retrospective design, which resulted in missing data in addition to the small cohort of patients. Another limitation of this study is that the cut-off value for diagnosing papillitis based on OCT-RNFLT was derived from the study by Kouwenberg et al.,7 in which a different OCT device was used (CIRRUS HD-OCT 5000 spectral domain OCT; Carl Zeiss Meditec) compared with the device used in our study (Heidelberg Spectralis OCT; Heidelberg Engineering, Heidelberg, Germany). Faghihi et al [24]. reported that RNFL thickness measurements obtained with the Spectralis OCT were higher than those obtained with the Cirrus OCT, with a mean difference of 4.67 ± 6.55 μm (p < 0.001). Similarly, Mitsch et al [25]. found a mean difference of 5.46 ± 1.1 μm between the two devices. Despite these findings, the inter-device difference in RNFL thickness is unlikely to be clinically significant in the present study, as the patients demonstrated markedly thickened RNFL values (mean 209.6 ± 88 μm).

However, the study’s strengths include focusing on a homogeneous group of children with pars planitis, a form of inflammation with distinct ocular characteristics not secondary to systemic disease. Other strengths are the long-term follow-up period and the ability to comprehensively assess both clinical and imaging features. Furthermore, OCT-RNFL measurements in our cohort were consistently obtained using a single OCT device, enhancing measurement reliability.

In conclusion, our findings suggest that OCT-RNFLT is a valuable imaging biomarker for assessing disease severity in children with pars planitis. As an objective and quantitative measure, OCT-RNFLT strongly correlates with disease activity. An OCT-RNFL thickness ≥ 130 µm indicates the need for a more aggressive immunosuppressive approach, which can help prevent structural ocular complications. The observed reduction in OCT-RNFLT with resolving inflammation highlights its potential role as a tool for monitoring therapeutic response over time. Prospective, multicenter studies are warranted to validate its predictive utility. To our knowledge, this is the first longitudinal study to investigate OCT-RNFL changes in pediatric pars planitis patients, providing detailed insights into disease progression and treatment response.

## Data Availability

The datasets used and/or analysed during the current study are available from the corresponding author on reasonable request.
